# Biological and clinical implications of hsa_circ_0086720 in gastric cancer and its clinical application

**DOI:** 10.1002/jcla.24369

**Published:** 2022-03-25

**Authors:** Yongfu Shao, Changlei Qi, Jianing Yan, Rongdan Lu, Guoliang Ye, Junming Guo

**Affiliations:** ^1^ Department of Gastroenterology The Affiliated Hospital of Medical School of Ningbo University Ningbo China; ^2^ Department of Biochemistry and Molecular Biology and Zhejiang Key Laboratory of Pathophysiology Ningbo University School of Medicine Ningbo China

**Keywords:** biomarker, CircRNA, gastric cancer, hsa_circ_0086720, prognosis

## Abstract

**Background:**

Circular RNAs (circRNAs) are thought to be vital participants in carcinogenesis and have the characteristics of being stable, specific, and well conserved. However, their clinical significance and application value in gastric cancer (GC) are still poorly understood. Hsa_circ_0086720 was found to be a dysregulated circRNA in GC by microarray screening and was further explored for its clinical significance and application.

**Methods:**

Hsa_circ_0086720 was detected in GC cell lines, tissues, and plasma, and the clinicopathological correlations were investigated. The existence, stability, origin, and change in the plasma hsa_circ_0086720 level were verified in early GC patients. Moreover, receiver operating characteristic and Kaplan–Meier survival curves were constructed to analyze the diagnostic and prognostic values, and bioinformatics analysis was used to identify the potential functions. Finally, risk factors and nomogram predicting were established.

**Results:**

Hsa_circ_0086720 was found to be downregulated in gastric carcinogenesis, and tissue hsa_circ_0086720 was negatively associated with perineural invasion, Borrmann type, disease‐free survival, and overall survival. Hsa_circ_0086720 was stable in circulating plasma and was actively secreted by cells in gastric carcinogenesis. As a biomarker for early GC screening, plasma hsa_circ_0086720 had good sensitivity and specificity, and its stability met the clinical application requirements. Bioinformatics analysis suggested that dysregulated hsa_circ_0086720 has important functions in gastric carcinogenesis. Univariate Cox regression analysis identified factors associated with overall survival time and disease‐free survival time. The nomograms showed good accuracy of predicting survival time.

**Conclusion:**

Hsa_circ_0086720 is a novel biomarker for screening early GC and predicting the prognosis of advanced‐stage patients.

## Graphical abstract

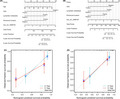

## 1 INTRODUCTION

Gastric cancer (GC) is the fifth most common cancer and the third leading cause of cancer‐related death.^1^ Although diagnostic and treatment technologies have greatly improved, the prognosis of GC patients is still unsatisfactory because of the high recurrence rate and low 5‐year overall survival (OS) rate.^2^, ^3^ Possible explanations for this phenomenon may include indistinct molecular mechanisms of GC progression and an extremely low early diagnostic rate.^4^ To date, early and advanced GC has still been mainly diagnosed by gastroscopy for symptomatic patients, and screening of clinically asymptomatic patients has been limited largely due to the absence of desirable biomarkers. Thus, the identification of new biomolecules for GC screening and treatment will be advantageous for reversing the current situation.

Distinctly different from linear RNAs, circular RNAs (circRNAs) have a special structure that lacks a 5′‐terminal cap and 3′‐terminal polyA tail, and the ends are joined together.^5^, ^6^, ^7^ Although circRNAs were previously deemed to be byproducts of the RNA splicing process, they have gradually been reclassified from “junk” to “treasure” with the help of next‐generation sequencing.^8^, ^9^ Recently, the critical functions of circRNAs have mainly been positioned to (1) act as miRNA sponges or transcriptional controllers to participate in gene regulation^8^; (2) be translated to proteins closely related to carcinogenesis^9^; (3) interact with RNA‐binding proteins to regulate cell function^8^, ^10^; and (4) stably exist in body fluids and meet clinical testing requirements.^11^, ^12^ These findings provide a new direction for circRNA research in carcinogenesis.

Abnormal expression profiles of circRNAs in GC tissues have been covered by circRNA microarrays in our previous studies.^13^ According to the microarray analysis results, we found that hsa_circ_0086720 is a significantly dysregulated circRNA in GC. Hsa_circ_0086720 contained 714 nucleotides in the final spliced sequence. Sequence analysis showed that its gene is located at human chr9:33953282–33996331 with a length of 43,049 nt, and the associated symbol is UBAP2 (ubiquitin‐associated protein 2).

In this study, hsa_circ_0086720 was chosen as a target to explore its clinical significance and potential applications in gastric carcinogenesis. Thus, the existence, stability, origin, change, clinical values, and potential functions of hsa_circ_0086720 were verified in GC patients. Our study indicates that hsa_circ_0086720 is a novel biomarker for early GC screening and prognostic estimation in advanced‐stage patients.

## 2 MATERIALS AND METHODS

### 2.1 Sample materials

A total of 96 GC tissues and paired adjacent nontumor tissues were obtained by surgical resection from GC patients, while 24 early GC (EGC) tissues and paired adjacent nontumor tissues were obtained by endoscopic submucosal dissection (ESD).^14^ EGC is generally defined as cancer cells confined to the mucosa or submucosa, regardless of tumor size or local lymph node metastasis. Peripheral blood was collected from 40 healthy volunteers and 42 EGC patients who were finally pathologically diagnosed with intramucosal adenocarcinoma after ESD. Blood was collected before ESD treatment. Plasma and tissues were stored at −80°C.^15^

The diagnosis of the patients was ultimately confirmed by pathology. The tumor‐node‐metastasis (TNM) staging system (7^th^ ed.) and National Comprehensive Cancer Network clinical practice guideline of oncology (V.1.2012) were used to determine the clinical stages and histological grades of tumors, respectively.^16^ Borrmann type is classified according to the tumor appearance. According to Borrmann type, advanced GCs were classified into the following four types: nodular fungus type (Ⅰ), localized ulcer type (Ⅱ), ulcer infiltration type (Ⅲ), and diffuse infiltration type (Ⅳ). Written informed consent was obtained from all patients.

### 2.2 Cell culture

Cell lines were purchased from the Chinese Academy of Sciences or Shanghai Institute of Biochemistry and Cell Biology. Cells were cultured in RPMI‐1640 medium (Life Technologies) supplemented with 10% fetal bovine serum (Life Technologies) at 37°C with 5% CO_2_.

### 2.3 Quantitative real‐time reverse transcription‐polymerase chain reaction detection

Tissue RNA was extracted using TRIzol (Ambion), while plasma was extracted using TRIzol LS reagent (Ambion). Total RNA was reverse transcribed to cDNA with a GoScript Reverse Transcription (RT) System (Promega). qRT‐PCR detection was conducted with GoTaq qPCR Master Mix (Promega). The qRT‐PCR conditions were as follows: 95°C for 5 min, followed by 45 cycles of 94°C for 15 s, 55°C for 30 s, and 72°C for 30 s. GAPDH mRNA was used to normalize circRNA expression. The primer sequences were as follows: hsa_circ_0086720: forward, 5′‐ACTGCCGTCAACTCCTGTTC‐3′, reverse, 5′‐TGTCTGAATTCCCTTCCAGCAA‐3′; GAPDH: forward, 5′‐ACCCACTCCTCCACCTTTGAC‐3′, reverse, 5′‐TGTTGCTGTAGCCAAATTCGTT‐3′. The hsa_circ_0086720 level was calculated using the Δ*C*t method (Δ*C*t = *C*t _hsa_circ_0086720_ ‐ *C*t_GAPDH_). A higher Δ*C*_t_ value means a lower hsa_circ_0086720 level.

### 2.4 Sequencing of hsa_circ_0086720 qRT‐PCR products

Hsa_circ_0086720 qRT‐PCR products were purified and cloned into the pUCm‐T vector (Sangon Biotech). Finally, sequencing was performed by Sangon Biotech (https://www.sangon.com/).

### 2.5 Bioinformatics analysis

MicroRNA (miRNA) interaction with hsa_circ_0086720 was predicted by Arraystar prediction software.^17^ Gene Ontology (GO) and Kyoto Encyclopedia of Genes and Genomes (KEGG) pathway analyses were performed with miRPath (http://diana.imis.athena‐innovation.gr). A network map was drawn by Cytoscape (https://cytoscape.org). The common downstream targets of miRNAs were displayed by Venny 2.1 (http://bioinfogp.cnb.csic.es/tools/venny/). *p* < 0.05 was used as the criterion for statistical significance.

### 2.6 Risk factors and nomogram predicting

Univariate Cox regression analyses were constructed to estimate the potential risk factors based on hsa_circ_0086720 levels and the clinicopathological parameters. Positive risk factors were selected and incorporated into prognostic nomogram predicting models. Calibration curves were used to validate the predictive performance of nomograms. Data were analyzed by R software package including “survival” and “rms.”

### 2.7 Statistical analysis

Statistical analysis was performed with Statistical Program for Social Sciences (SPSS) 20.0 Software (SPSS). Data were analyzed by Student's *t* test, one‐way ANOVA, or Kaplan–Meier analysis according to actual conditions. The statistical significance level was regarded as *p* < 0.05.

## 3 RESULTS

### 3.1 Hsa_circ_0086720 is decreased during carcinogenesis

**FIGURE 1:**
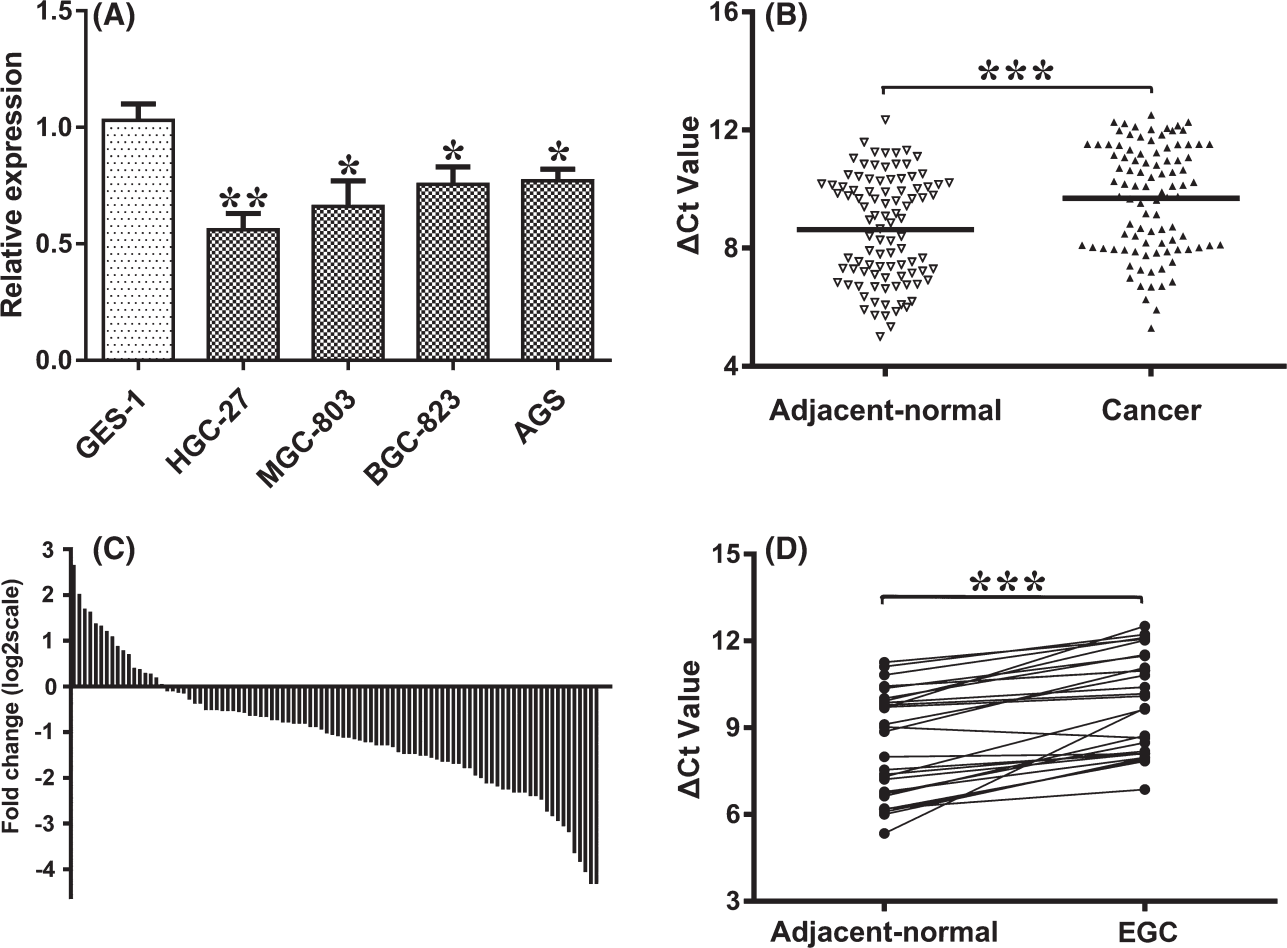
Hsa_circ_0086720 expression is downregulated during gastric carcinogenesis. (A) Hsa_circ_0086720 expression levels in four gastric cancer cell lines (AGS, BGC‐823, HGC‐27, and MGC‐803) and a normal gastric epithelial cell line (GES‐1) were determined by qRT‐PCR. (B) The expression levels of hsa_circ_0086720 in cancer tissues (n = 96) and adjacent normal tissues (n = 96). (C) Compared to expression in adjacent normal tissues, Hsa_circ_0086720 expression was significantly downregulated in 82.3% (79/96) of gastric cancer tissues. (D) Hsa_circ_0086720 expression was significantly downregulated in early‐stage EGC (n = 24). Data are shown as the means ± SDs of two independent experiments. A higher Δ*C*_t_ value means a lower hsa_circ_0086720 level. Asterisks represent significant differences (*p < 0.05, **p < 0.01, ***p < 0.001)

To verify the authenticity of hsa_circ_0086720 expression in the circRNA microarray, we detected expression in cell lines and tissues. Compared to expression in the normal gastric epithelial cell line GES‐1, hsa_circ_0086720 expression in GC cells was significantly downregulated (Figure 1A). Consistent with the cell results, compared to expression in control tissues, hsa_circ_0086720 expression was significantly decreased in 82.3% (79/96) of GC tissues (*p* < 0.001, Figure 1B,C).

Next, we explored hsa_circ_0086720 expression in 24 paired EGC tissues collected from ESD patients. Similar to the GC results, hsa_circ_0086720 was also downregulated in the very early stage of EGC (*p* < 0.001, Figure 1D).

### 3.2 Clinicopathological correlation analysis

Hsa_circ_0086720 in GC tissue was significantly negatively associated with Borrmann type (*p* < 0.001) and perineural invasion (*p* = 0.029). However, we did not find other clinicopathological correlations, such as distal metastasis, differentiation, and TNM stage (Table 1).

**TABLE 1:** Relationship of hsa_circ_0086720 expression levels (Δ*C*_t_) in cancer tissues with clinicopathological factors of GC patients

Characteristics	No. of case (%)	Mean ± SD	*p* value
Age (years)			
≥60	61 (63.5)	9.936 ± 1.837	0.083
<60	35 (36.5)	9.266 ± 1.742	
Gender			
Male	65 (67.7)	9.738 ± 1.916	0.721
Female	31 (32.3)	9.595 ± 1.634	
Tumor location			
Sinuses ventriculi	49 (51.1)	9.824 ± 1.641	0.549
Cardia	10 (10.4)	9.578 ± 2.665	
Corpora ventriculi	25 (26.0)	9.283 ± 1.750	
Others	12 (12.5)	10.097 ± 1.950	
Diameter (cm)			
≥5	47 (49.0)	9.757 ± 1.854	0.734
<5	49 (51.0)	9.629 ± 1.809	
Differentiation			
Well	12 (12.5)	8.896 ± 1.859	0.270
Moderate	47 (49.0)	9.831 ± 1.884	
Poor	37 (38.5)	9.772 ± 1.711	
Stage			
Early	24 (25.0)	9.533 ± 1.567	0.626
Advanced	72 (75.0)	9.744 ± 1.907	
Borrmann type			
I&II	19 (26.4)	11.056 ± 1.395	<0.001
III&IV	53 (73.6)	9.274 ± 1.854	
Pathologic diagnosis			
Signet ring cell cancer	15 (15.6)	9.817 ± 1.613	0.774
Adenocarcinoma	81 (84.4)	9.668 ± 1.867	
Invasion			
T_1_&T_2_	36 (37.5)	9.792 ± 1.853	0.678
T_3_&T_4_	60 (62.5)	9.631 ± 1.817	
Lymphatic metastasis			
N_0_	38 (39.6)	9.830 ± 1.699	0.550
N_1‐3_	58 (60.4)	9.601 ± 1.908	
Distal metastasis			
M_0_	82 (85.4)	9.755 ± 1.801	0.415
M_1_	14 (14.6)	9.322 ± 1.970	
Venous invasion			
Absent	53 (55.2)	9.906 ± 1.697	0.201
Present	43 (44.8)	9.427 ± 1.954	
Perineural invasion			
Absent	47 (49.0)	10.105 ± 1.850	0.029
Present	49 (51.0)	9.295 ± 1.722	
CEA(Tissue)			
Positive	74 (77.1)	9.570 ± 1.716	0.231
Negative	22 (22.9)	10.102 ± 2.134	
CA19‐9 (Tissue)			
Positive	54 (56.3)	9.572 ± 1.832	0.469
Negative	42 (43.7)	9.845 ± 1.820	

### 3.3 Prognostic value of hsa_circ_0086720 in GC

**FIGURE 2:**
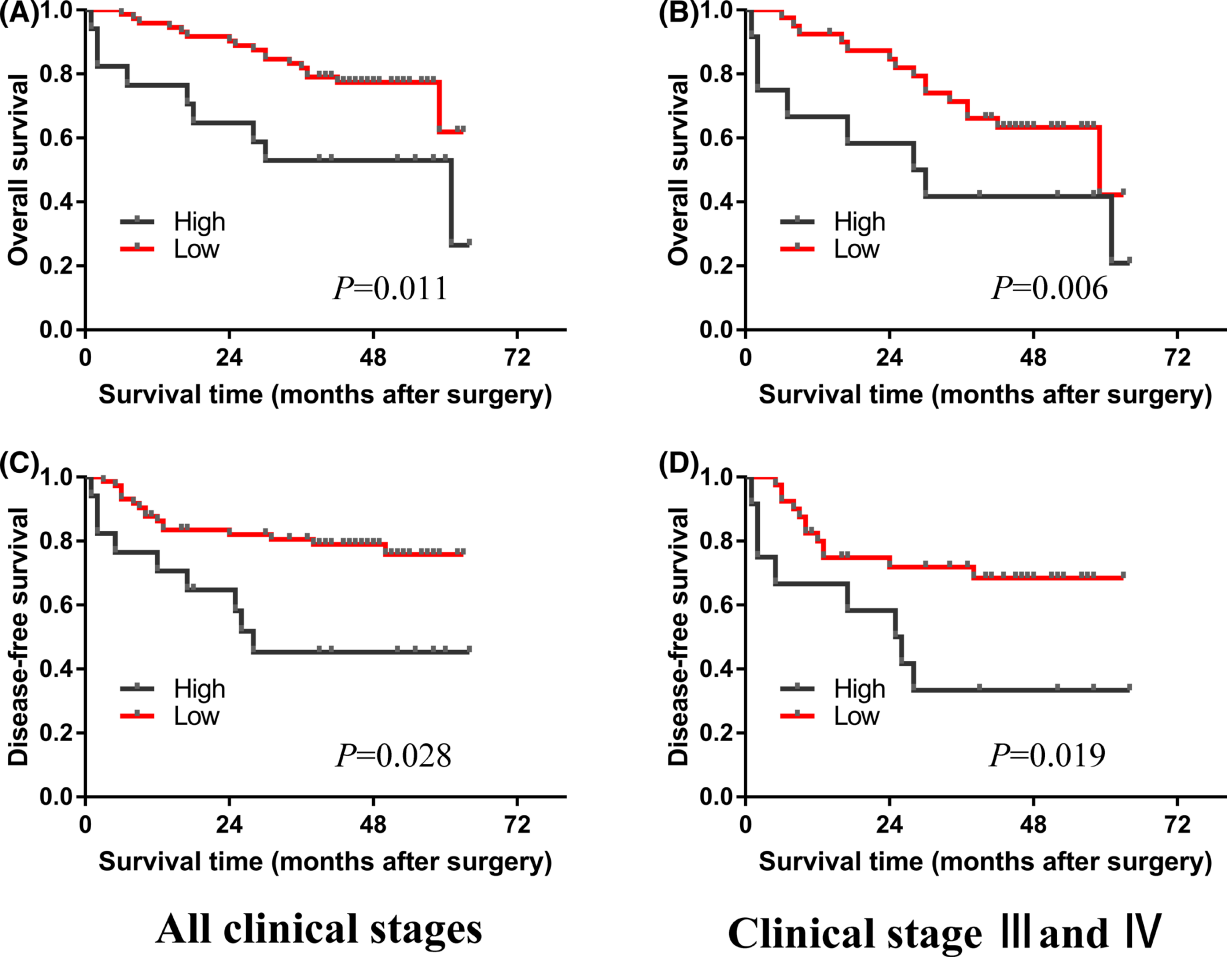
Kaplan–Meier survival plot. Patients in the low hsa_circ_0086720 expression group had longer overall survival (OS) and disease‐free survival (DFS) times than those in the high expression group; this trend was not only confined to advanced clinical stages (stages III and IV; B, D) but also occurred in all clinical stages (A, C)

Surgical patients were divided into "low" or "high" groups according to their hsa_circ_0086720 levels in GC tissues compared to the levels in paired adjacent nontumor tissues. The ΔΔ*C*t method (ΔΔ*C*t = Δ*C*t _GC tissue_–Δ*C*t _adjacent nontumor tissues_) was used to compare the hsa_circ_0086720 levels in GC tissues and paired adjacent nontumor tissues. The actual cutoff point was 0. If the patient's ΔΔ*C*t value was greater than 0, they were assigned to the low expression group; otherwise, they were assigned to the high expression group. Then, Kaplan–Meier analysis was performed to distinguish the differences in OS and disease‐free survival (DFS) between the "low" and "high" hsa_circ_0086720 expression level groups. Interestingly, GC patients in the low group had longer OS and longer DFS times than those in the high group, and this trend was not only confined to advanced cancer stages (stages III and IV; Figure 2B,D) but also applied to all clinical stages (Figure 2A,C).

### 3.4 Clinical diagnostic values as a biomarker

**FIGURE 3:**
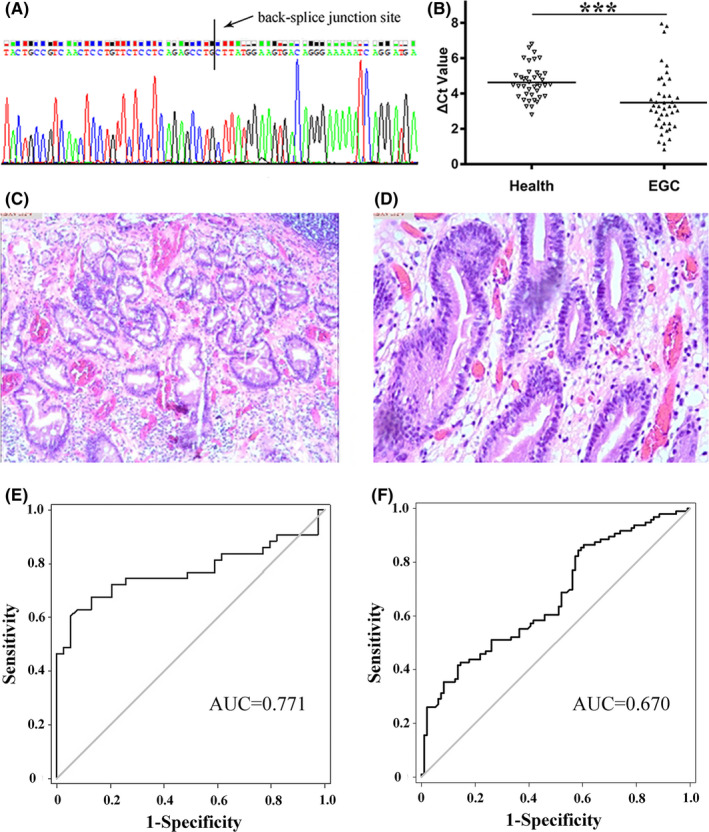
Clinical diagnostic value of hsa_circ_0086720 as a biomarker. (A) DNA sequencing results of plasma hsa_circ_0086720. The qRT‐PCR products of hsa_circ_0086720 were sequenced. Primer amplification sequences contain the back‐splice junction site. (B) Hsa_circ_0086720 levels in the plasma of patients in the EGC stage (n = 40) were increased significantly compared to those in healthy controls (n = 42). (C, D) EGC patients were finally pathologically diagnosed with intramucosal adenocarcinoma designated as locally cancerous. (E) ROC curve of plasma hsa_circ_0086720 as a biomarker for EGC screening. The ROC curve was up to 0.771 (95% CI, 0.663–0.879; *p* < 0.001). (F) ROC curve of hsa_circ_0086720 in differentiating gastric cancer tissues from controls. The AUC of hsa_circ_0086720 in cancer tissues was only up to 0.670 (95% CI, 0.594–0.746; *p* < 0.001). Data are two independent experiments. A higher Δ*C*_t_ value means a lower hsa_circ_0086720 level. Asterisks represent significant differences (***p < 0.001)

Circulating plasma is a suitable material for early cancer screening in clinical practice. We wondered whether hsa_circ_0086720 might exist in circulating plasma. Thus, the plasma PCR products were sequenced. As expected, the sequencing results were consistent with the sequence in circBase (http://circrna.org/; Figure 3A). Moreover, primer amplification sequences contain the back‐splice junction site (Figure 3A). The DNA sequence confirmed the existence of circulating plasma hsa_circ_0086720.

Then, circulating plasma from 40 healthy volunteers and 42 EGC patients was obtained and quantified by qRT‐PCR. As shown in Figure 3B, the plasma hsa_circ_0086720 levels were significantly increased in the EGC stage (*p* < 0.001). EGC patients were finally pathologically diagnosed with intramucosal adenocarcinoma and were designated as locally cancerous (Figure 3C‐D). Moreover, it is noteworthy that the changes in hsa_circ_0086720 in plasma and cancer tissue were completely opposite.

Next, an ROC curve was constructed to investigate the potential value of plasma hsa_circ_0086720 in EGC screening. We found that the area under the ROC curve (AUC) was up to 0.771 (95% confidence interval [CI], 0.663–0.879; *p* < 0.001; Figure 3E). The cutoff value, sensitivity, and specificity were 3.77, 67.4%, and 87.2%, respectively (Figure 3E). However, the AUC of hsa_circ_0086720 in cancer tissues was up to only 0.670 (95% CI, 0.594–0.746; *p* < 0.001; Figure 3F), which was lower than that in plasma. In cancer tissue, the sensitivity and specificity of hsa_circ_0086720 were 41.67% and 86.46%, respectively. Compared to tissue hsa_circ_0086720, plasma hsa_circ_0086720 had a higher diagnostic value.

### 3.5 Plasma hsa_circ_0086720 is mainly derived from cells

**FIGURE 4:**
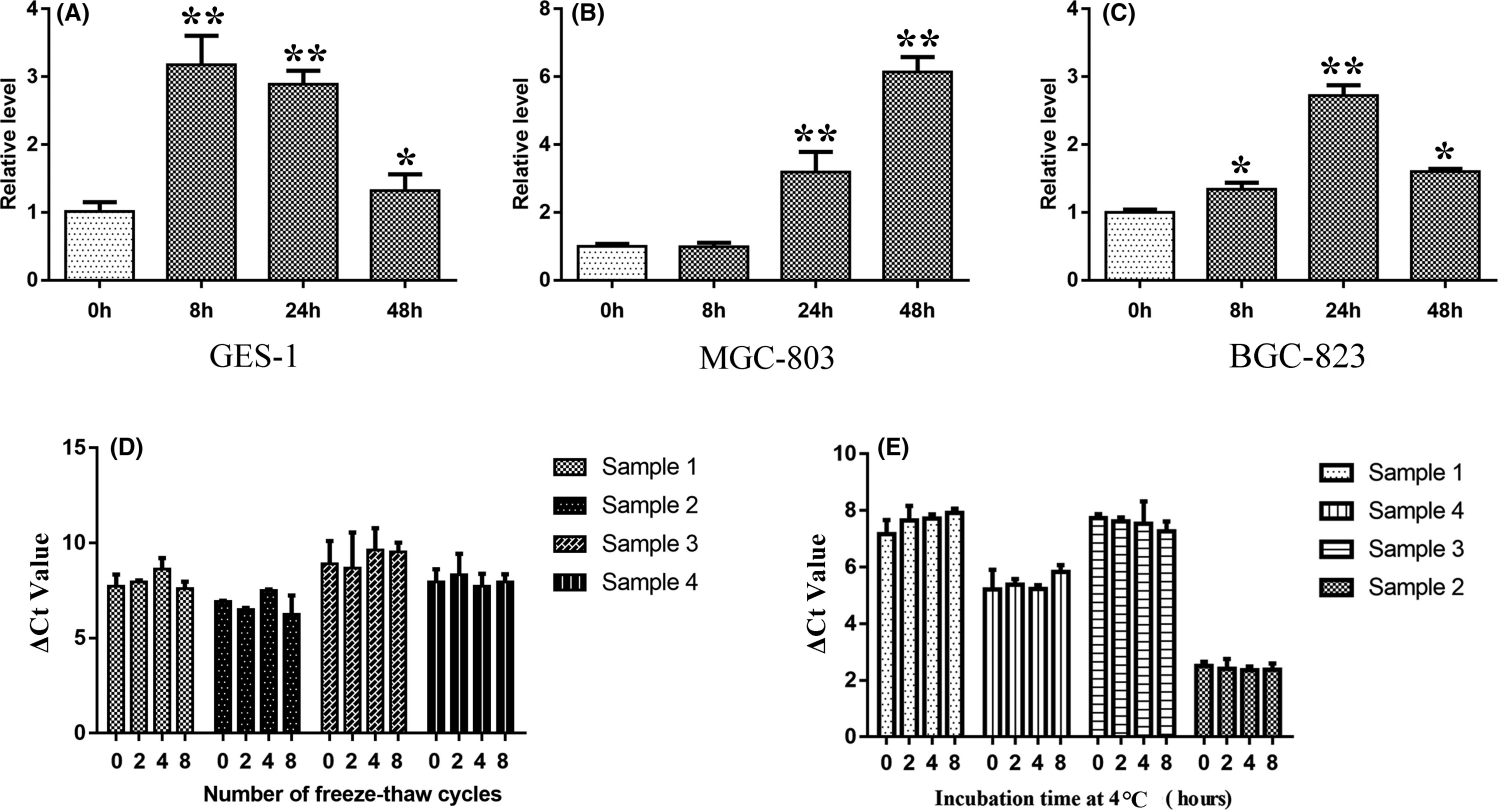
The origin and stability of hsa_circ_0086720 in plasma. (A‐C) Gastric cell culture experiments. The normal human gastric mucosa epithelial cell line GES‐1 and gastric cancer cell lines BGC‐823 and MGC‐803 were cultured in serum‐free medium. qRT‐PCR was used to detect the hsa_circ_0086720 levels in medium after 0, 8, 24, and 48 h of incubation. *p < 0.05, **p < 0.01. (D) Results of freeze‐thaw experiments (*p* > 0.05). Four blood samples were randomly selected and then equally divided into four parts. After 0, 2, 4, and 8 freeze‐thaw cycles, the hsa_circ_0086720 levels were detected. (E) Results of the incubation experiments (*p* > 0.05). Four blood samples were randomly selected and then equally divided into four parts and placed at 4 °C. After 0, 2, 4, and 8 h of incubation, the hsa_circ_0086720 levels were detected

Gastric cells were cultured in serum‐free medium, and the supernatant hsa_circ_0086720 was measured after 0, 8, 24, and 48 h of incubation. As expected, hsa_circ_0086720 in the cell supernatant tended to increase in both normal gastric cells and cancer cells (Figure 4A‐C; *p* < 0.05).

### 3.6 The stability of hsa_circ_0086720 in plasma

Good stability is a prerequisite for clinical detection. To test whether the stability of plasma hsa_circ_0086720 meets clinical requirements, freeze‐thaw and incubation experiments were performed. Our results confirmed that plasma hsa_circ_0086720 had good stability within a limited number of freeze‐thaw and incubation times, which meets the needs of routine clinical detection (Figure 4D,E).

### 3.7 Prediction for hsa_circ_0086720 function

**FIGURE 5:**
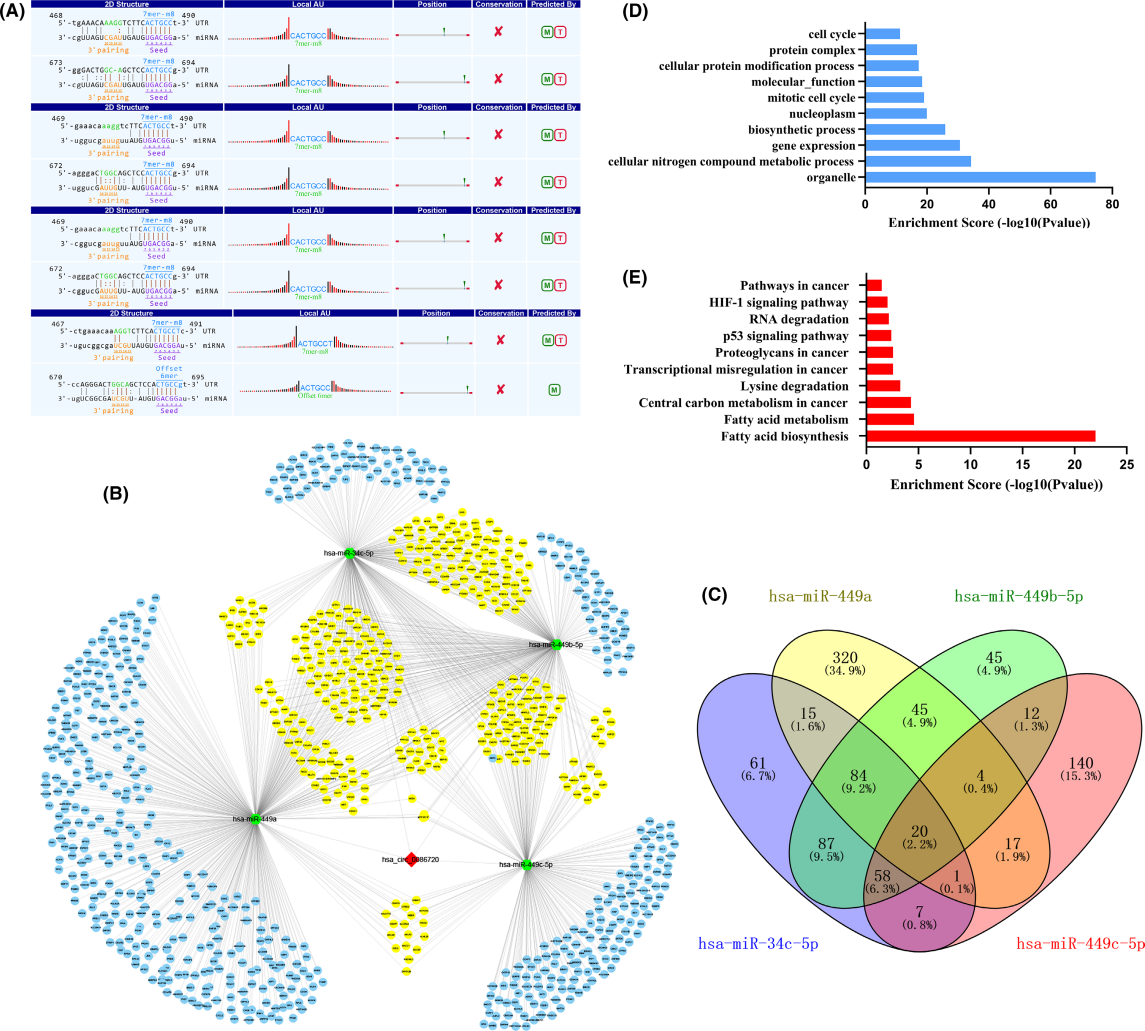
Prediction of hsa_circ_0086720 function. (A) Prediction of hsa_circ_0086720/miRNA interactions. (B) A network map showing hsa_circ_0086720, four miRNAs, and their downstream targets is presented. (C) Venn diagram revealing the number of common downstream targets of four miRNAs. (D) The hsa_circ_0086720‐miRNA axes related to GO analysis. (E) The hsa_circ_0086720‐miRNA axes related to KEGG pathway analysis

Hsa_circ_0086720 was predicted to harbor the hsa‐miR‐34c‐5p, hsa‐miR‐449a, hsa‐miR‐449b‐5p, and hsa‐miR‐449c‐5p seed sequences (Figure 5A). Hsa_circ_0086720‐related miRNAs and the hsa_circ_0086720‐miRNA axes are presented in Figure 5B. GO and KEGG pathway analyses showed that the hsa_circ_0086720‐miRNA axis was involved in various biological functions and signaling pathways (Figure 5D,E).

### 3.8 Predictors selection and nomogram model development

**FIGURE 6:**
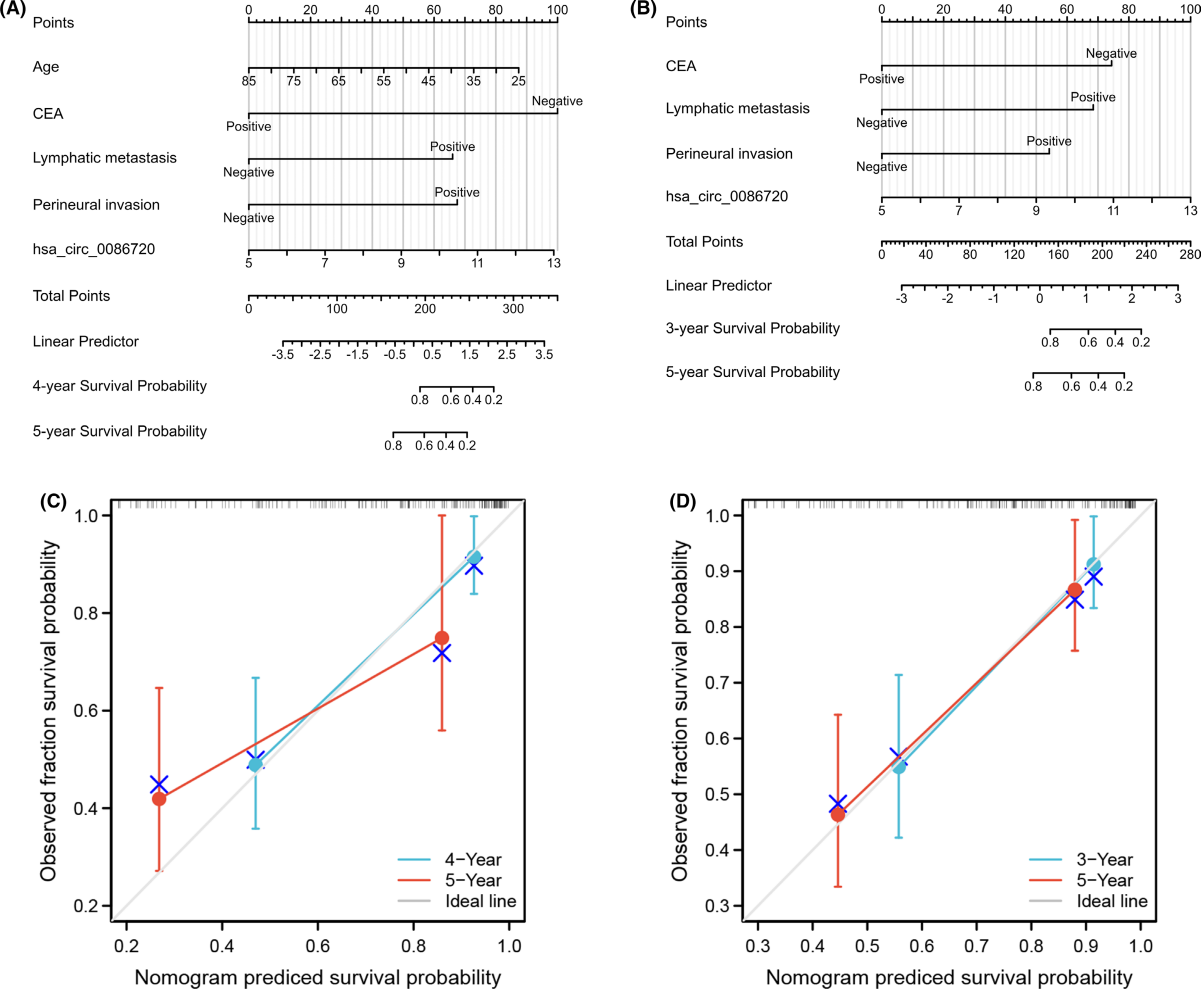
Nomogram for risk assessment of survival time. (A, B) The nomograms showed good accuracy in predicting OS (C‐index, 0.822) and DFS (C‐index, 0.772). (C, D) Calibration curves reflected good discriminative ability of prognosis

Univariate Cox regression analysis were constructed to estimate the potential risk factors associated with OS time and DFS time based on hsa_circ_0086720 levels and the clinicopathological parameters. As shown in Table 2, Age, CEA, lymphatic metastasis, perineural invasion, and hsa_circ_0086720 level were associated with OS, whereas CEA, lymphatic metastasis, perineural invasion, and hsa_circ_0086720 level were independently related with DFS (Table 2). Positive risk factors were selected and incorporated into prognostic nomogram predicting models. The nomograms showed good accuracy of predicting OS (C‐index, 0.822; Figure 6A) and DFS (C‐index, 0.772; Figure 6B). Calibration curves reflected good discriminative ability of prognosis (Figure 6C,D).

**TABLE 2:** Univariate Cox regression analysis of survival time

Clinical Variable	Overall survival time		Disease‐free survival time	
	Hazard ratio (95% C.I.)	*p* value	Hazard ratio (95% C.I.)	*p* value
Sex	0.757	0.577	0.973	0.955
Age	0.935	0.001	0.972	0.165
CEA	0.047	0.001	0.164	0.001
CA19‐9	0.581	0.308	0.528	0.196
Lauren's type	0.411	0.289	0.525	0.393
Distal metastasis	3.545	0.071	0.935	0.931
Venous invasion	2.564	0.078	1.424	0.460
Lymphatic metastasis	4.766	0.030	7.373	0.006
Perineural invasion	4.821	0.017	4.092	0.018
Differentiation	0.827	0.770	1.072	0.915
Diameter	1.044	0.668	1.004	0.968
TNM stage	1.189	0.633	0.917	0.812
Hsa_circ_0086720	1.566	0.003	1.480	0.005

Note

Entry 0.05; Removal: 0.1.

## 4 DISCUSSION

The rapid development of biological technologies has contributed to the in‐depth exploration of functional circRNAs.^17^ CircRNAs are characterized as being stable, specific, and well conserved and have been suggested to be vital participants in carcinogenesis, making them possible candidates for cancer screening.^18^, ^19^, ^20^ Accordingly, circRNAs have attracted more attention in tumor pathogenesis and biomarker research in recent years. In the field of GC, a number of abnormally expressed circRNAs have been discovered, and their functions and molecular mechanisms have been revealed.^13^, ^21^ For example, Yu et al.^22^ reported that circ‐TNPO3 acts as a protein decoy for IGF2BP3 to regulate the MYC‐SNAIL axis suppressing the proliferation and metastasis of GC. Zhang et al.^23^ identified that circLARP4 could be an miR‐424‐5p sponge and regulate large tumor suppressor kinase 1 (LATS1) expression to affect cell proliferation and invasion of GC. All this evidence suggests that circRNAs are emerging molecular targets for GC screening, diagnosis, and treatment.

Hsa_circ_0086720 is a dysregulated circRNA measured by microarray analysis in our previous studies.^13^ In the current study, we found that compared to expression in controls, hsa_circ_0086720 expression was decreased in all four GC cell lines and in 82.3% of GC tissues (Figure 1A‐C). More importantly, hsa_circ_0086720 was also significantly downregulated at the very early stage of EGC (Figure 1D). Bioinformatics analysis revealed that hsa_circ_0086720 harbors some miRNA seed sequences and that the hsa_circ_0086720‐miRNA axis is involved in various biological functions and signaling pathways (Figure 5). This indicated that low hsa_circ_0086720 expression in gastric tissues is closely associated with the initiation and development of gastric carcinogenesis.

Recent studies have confirmed that several important clinicopathological features are independent prognostic factors of GC.^24^, ^25^, ^26^ Luo et al.^24^ conducted a meta‐analysis to obtain the results that Borrmann type IV GC was associated with lymph node metastases, poor cell differentiation, and poor prognosis. Li et al.^25^ confirmed that Borrmann type was a worthy survival predictor for advanced GC patients. Similar to the Borrmann type, perineural invasion (PNI) appeared to be an independent prognostic factor for OS of GC patients with positive PNI, and PNI is valuable for detecting patients with a poor prognosis.^26^ This information suggests that molecules have the potential to predict GC prognosis if their expression is related to Borrmann type and perineural invasion. In our study, hsa_circ_0086720 in cancer tissues was associated with Borrmann type and perineural invasion (Table 1). Moreover, GC patients with lower hsa_circ_0086720 levels had longer OS and longer DFS times in all clinical stages (Figure 2). Our data show that hsa_circ_0086720 has prognostic value for GC patients.

Plasma detection is an effective method for screening early cancer in the clinic. However, the classic blood biomarkers currently used in the clinic do not have satisfactory sensitivity and specificity. For EGC screening, the positive rates of the carcinoembryonic antigen (CEA), carbohydrate antigens19–9 (CA19‐9), and cancer antigen 125 (CA125) levels were 4.3%, 4.8%, and 1.9%, respectively.^27^ The highest positive rate was only 10.4% for the combination of all three biomarkers in EGC.^27^ Early cancer screening by blood biomarkers still faces challenges. In this study, we wondered whether hsa_circ_0086720 could exist in human circulating plasma and be used as an early GC screening biomarker. As expected, we confirmed its existence in plasma by cloning and sequencing methods (Figure 3A). PCR results identified that the levels of plasma hsa_circ_0086720 were significantly increased in the EGC stage (Figure 3B). The ROC curve further confirmed the potential values of plasma hsa_circ_0086720 in EGC screening (Figure 3E). As a screening biomarker, the sensitivity and specificity of hsa_circ_0086720 for EGC were 67.4% and 87.2%, respectively. This means that compared to biomarkers currently used in the clinic, hsa_circ_0086720 has better sensitivity and specificity. Combined with the existing markers, endoscopy and hsa_circ_008672 may greatly improve the detection rate of EGC and make up for the diagnostic deficiencies of a single marker. Moreover, freeze‐thaw and incubation experiments confirmed the stability of plasma hsa_circ_0086720, which implies that it meets the requirements needed for routine clinical detection (Figure 4D,E). Therefore, hsa_circ_0086720 is a potential marker for early GC screening.

Exosomes are 30 to 150 nm endocytic membrane‐derived vesicles actively secreted by cells.^28^ Tumor‐derived exosomes have reportedly been involved in carcinogenesis and can function as diagnostic biomarkers for specific cancers.^28^, ^29^ Recent research has shown that nucleic acids can be highly enriched and selectively released by exosomes to participate in cell communication.^30^, ^31^, ^32^ Currently, in this study, the abnormal phenomenon of the opposite changes in the plasma and cancer tissue hsa_circ_0086720 levels has aroused our great interest. Thus, we speculated that plasma hsa_circ_0086720 is actively secreted by exosomes during GC carcinogenesis. As expected, we preliminarily verified that hsa_circ_0086720 in the supernatant tended to increase with time (Figure 4A‐C). This result indicates that plasma hsa_circ_0086720 mainly exists in exosomes and is actively secreted by cells during gastric carcinogenesis. In addition, the existing form of hsa_circ_0086720 also gives it better stability in plasma.

In this study, we chose hsa_circ_0086720 as a targeted circRNA to explore its clinical significance in gastric carcinogenesis. Our study validated the existence of hsa_circ_0086720 and analyzed the potential relationship between this circRNA and GC clinicopathological factors in 96 patients. However, we did not find other clinicopathological correlations, such as distal metastasis, differentiation, and TNM stage. This does not mean that our data completely negate the potential relationship between hsa_circ_0086720 and other pathological factors. If the sample size is increased, other potential correlations may appear. Therefore, more samples are needed in future studies, and the biological function of hsa_circ_0086720 in cancer cells needs further experimental verification.

In conclusion, our study indicates that hsa_circ_0086720 is a novel biomarker for early GC screening and prognostic estimation in advanced‐stage patients.

## CONFLICT OF INTEREST

The authors disclose no conflict.

## Data Availability

The data that support the findings of this study are available from the corresponding author upon reasonable request.
